# General and specific consciousness: a first-order representationalist approach

**DOI:** 10.3389/fpsyg.2013.00407

**Published:** 2013-07-16

**Authors:** Neil Mehta, George A. Mashour

**Affiliations:** ^1^Whitney Humanities Center, Yale UniversityNew Haven, CT, USA; ^2^Neuroscience Graduate Program, Department of Anesthesiology, University of Michigan Medical SchoolAnn Arbor, MI, USA

**Keywords:** consciousness, neural correlate, level of consciousness, content of consciousness, representationalism, first-order representationalism

## Abstract

It is widely acknowledged that a complete theory of consciousness should explain general consciousness (what makes a state conscious at all) and specific consciousness (what gives a conscious state its particular phenomenal quality). We defend first-order representationalism, which argues that consciousness consists of sensory representations directly available to the subject for action selection, belief formation, planning, etc. We provide a neuroscientific framework for this primarily philosophical theory, according to which neural correlates of general consciousness include prefrontal cortex, posterior parietal cortex, and non-specific thalamic nuclei, while neural correlates of specific consciousness include sensory cortex and specific thalamic nuclei. We suggest that recent data support first-order representationalism over biological theory, higher-order representationalism, recurrent processing theory, information integration theory, and global workspace theory.

The last two decades have witnessed a renaissance of scientific inquiry into consciousness. Several theoretical contenders have emerged, including *biological theory*, *higher-order representationalism*, *recurrent processing theory*, *information integration theory*, and *global workspace theory*. This article outlines another proposal, *first-order representationalism*, which has been influential among philosophers but has received scant neuroscientific attention. In this article, we state the testable differences between first-order representationalism and its rivals, and argue that current data favor first-order representationalism.

The core idea of first-order representationalism is that any conscious state is a *representation*, and what it’s like to be in a conscious state is wholly determined by the *content* of that representation. By definition, a representation is about something, and the content of a representation is what the representation is about. For instance, the word “dolphins” (representation) is about dolphins (content); Herbert Bix’s book *Hirohito and the Making of Modern Japan* (representation) is about the life of the Japanese emperor Hirohito (content); and the classic fairy tale Snow White (representation) is about the princess Snow White and an evil queen who tries to kill her (content). To express the first-order representationalist view in a (helpful but simplified) slogan: consciousness is the story that our perceptual systems tell us about the world. This is, of course, a substantial and controversial hypothesis, as many would dispute that our perceptual systems genuinely represent the world to us in this rich sense (Travis, 2004).

Three clarifications are in order. First clarification: though a representation has content, a representation is not identical to its content. For example, the representation “dolphins” is an English word with eight letters, but its content – dolphins – does not have any letters. Conversely, dolphins swim, but the word “dolphins” does not swim. This point underlies one major philosophical motivation of representationalism: the fact that neural states seem to have very different properties than conscious perceptions. For instance, when someone consciously perceives the color orange, normally there is nothing orange in that person’s brain. First-order representationalists explain this by holding that a conscious perception of orange is a representation of orange, and (as the “dolphin” example shows) the properties of a representation can be very different from the properties of its content (Dretske, 1995, 1999, 2003; Tye, 1995, 2000; Lycan, 1996).

Second clarification: the content of a representation can be false, and can concern a non-existent thing. Though Bix’s biography of Hirohito is carefully researched, doubtless it contains some false sentences. More to the point, the story of Snow White is about someone who does not exist. According to representationalists, this explains why illusions, dreams, and hallucinations are possible: consciousness can misrepresent the world.

Third clarification: first-order representationalism does not hold that every contentful representation is conscious. For example, early vision may contain unconscious representations (perhaps with contents concerning line orientations and light gradients), and the New York Times newspaper contains many representations that are not conscious. Thus, Tye (1995) suggests that a conscious representation must be poised to interact directly with one’s beliefs and desires, and similarly Dretske (2006) hypothesizes that a conscious representation must be available to the subject as a reason for action and belief formation. Below, we will refine these ideas from Tye and Dretske.

Stated at this high-level of abstraction, first-order representationalism delivers little in the way of scientifically testable predictions. This article explores a neuroanatomical interpretation of first-order representationalism that delivers testable predictions separating it from rival scientific theories. In doing so, we present a novel approach to the neural correlates of consciousness (defined below) that organizes general and specific consciousness in a functional relationship of post-sensory and sensory systems.

The discussion will proceed as follows. §1 distinguishes general consciousness (which pertains to the “level” of consciousness) from specific consciousness (which pertains to the “content” of consciousness) and also characterizes the post-sensory and sensory systems that participate in conscious processing. §2 summarizes several influential theories of consciousness and categorizes them according to which of these systems are hypothesized to be the neural correlates of general and specific consciousness. We argue that only first-order representationalism identifies post-sensory systems as the neural correlates of general consciousness and sensory systems as the neural correlates of specific consciousness. §3 argues that post-sensory systems are the neural correlates of general consciousness, while §4 argues that sensory systems are the neural correlates of specific consciousness, just as first-order representationalism predicts.

## 1. FRAMING THE PROBLEM(S) OF CONSCIOUSNESS

Let us begin by clarifying the phenomenon to be explained: consciousness. According to Block (1995, 2007), scientists and philosophers discussing “consciousness” have often conflated two quite different properties: *access consciousness* and *phenomenal consciousness*. To a first approximation, a mental state is access conscious if and only if one can directly and spontaneously use its content in action (including speech) and reasoning. By contrast, a mental state is phenomenally conscious if there is “something it’s like,” in the influential phrase of Nagel (1974), to be in that state. This paper focuses primarily on phenomenal consciousness, though issues of access consciousness crop up occasionally. In order to avoid confusion, we will always use the term “consciousness” to refer to phenomenal consciousness, and we will speak of “access” instead of “access consciousness.” Additionally, we will use the term *phenomenal character* to refer to “what it’s like” to be in a given conscious state.

Several theorists (Rees et al., 2002; Hurley and Noë, 2003; Tononi, 2004; Kriegel, 2009) have observed that a complete theory of consciousness must explain two separate phenomena. The first is what we term *general consciousness*: what makes a state conscious at all, as opposed to wholly unconscious. This part of a given theory should explain the difference between, e.g., one’s conscious state when one sees a red thing and one’s unconscious state when one is anesthetized.

Second, the theory must explain what we term *specific consciousness*: what gives a state its specific phenomenal character, rather than some alternative phenomenal character. This part of a given theory should explain the difference between one’s conscious state when one sees something red and one’s conscious state when one sees something green (or hears a loud noise, or feels pain). Specific consciousness presupposes general consciousness, since there can be no specific conscious state if the subject is not conscious at all. However, two individuals may both have general consciousness while differing in their specific consciousness.

To further elucidate the relationship between general consciousness and specific consciousness, consider an analogy. *Pride and Prejudice* is a book by Jane Austen. Certain factors make it a book at all – the fact that it has pages, a front and back cover, a binding, etc. Other factors make it the specific book that it is – the fact that it contains specific words in a specific order, was written by Jane Austen, etc. Similarly, general consciousness is what makes a state conscious at all, while specific consciousness is what makes a state the specific conscious state that it is.

This distinction between general and specific consciousness corresponds roughly to the existing distinction between the “level” and the “content” of consciousness (Hohwy, 2009; Overgaard and Overgaard, 2010; Bachmann, 2012). However, since we are using the term “content” in the technical way defined above, we will continue to use the general/specific terminology instead. Additionally, we acknowledge that cortical arousal, or the capacity for cortical arousal – which is mediated primarily by subcortical structures in the brainstem, diencephalon, and basal forebrain – is a necessary precondition for consciousness. However, these subcortical structures do not constitute consciousness *per se*. This is clearly evidenced in certain pathological conditions such as vegetative states, in which arousal or sleep–wake patterns can be present in the absence of consciousness (Laureys, 2005). Thus, we do not discuss these subcortical structures further.

Scientists have often sought the *neural correlates* of consciousness – the set of neural events and mechanisms that directly constitute (general or specific) consciousness. These differ from the *neural prerequisites* of consciousness, which are necessary for consciousness without directly constituting it, and from the *neural consequences* of consciousness, which result from consciousness rather than directly constituting it (Aru et al., 2012; De Graaf et al., 2012).

Broadly speaking, two types of systems participate in conscious processing. *Sensory systems* are dedicated to the detection of highly specific perceptible features. These systems may be tuned to modality-specific properties (such as color and tone) or properties detectible via multiple modalities (such as motion and spatial location), and the same localized neural region may be tuned to different perceptible properties over time (Lamme and Roelfsema, 2000). *Post-sensory systems* perform a broad variety of functions, including modulation of sensory processing via top-down attention, distribution of sensory information via a global workspace (Dehaene and Changeux, 2011), and estimation of signal quality (Lau, 2008).

For example, consider visual processing, which includes both a fast *feedforward sweep* and slower *recurrent processing* loops (also known as feedback, reentrant, reafferent, or reverberant processing; Lamme and Roelfsema, 2000; Lamme, 2006, 2010; Treisman, 2006; VanRullen, 2007; Dehaene et al., 2011). In the feedforward sweep, which is completed in about 100 ms, activation proceeds in a swift, unidirectional cascade from the retina, to the lateral geniculate nucleus of the thalamus, to V1 in the occipital cortex, to higher visual processing areas (including V2, V3, V4, and V5), and finally to more rostral structures. By contrast, recurrent processing loops involve reciprocal information transfer between these cortical areas, and corresponding thalamic regions (Jones, 2009), via horizontal and feedback connections. Recurrent processing may occur at many scales, from a local scale (within a sensory modality) to a global scale (implicating executive function or spanning different sensory modalities).

Plausibly, visual sensory systems dedicated to processing highly specific properties include occipital and temporal cortex (such as V1, V2, V3, V4, and V5), as well as the lateral geniculate nucleus of the thalamus. By contrast, many areas in the frontal and posterior parietal cortex, as well as thalamic non-specific (or matrix) nuclei associated with such areas (Jones, 2009), are strong candidates for post-sensory systems. These post-sensory systems tend to be multimodal association cortices that serve as highly connected hubs performing a wide range of functions (Gong et al., 2009).

Although the sensory/post-sensory distinction may not be perfectly sharp, we believe that it is sharp enough to be helpful in philosophical and scientific categorization. Consequently, the next section uses this framework to categorize theories of consciousness according to which systems (sensory or post-sensory) are hypothesized to be the neural correlates of general or specific consciousness. The commitments of scientific theories of consciousness with respect to this framework have usually been implicit. As such, we will make them as explicit as possible, which requires some degree of interpretation.

## 2. THEORIES OF CONSCIOUSNESS

It is widely accepted that consciousness is realized in neural states. However, is consciousness also identical to neural states, so that something without neural states (such as a robot) cannot possibly have the same kinds of conscious states as a human being? The *biological theory* answers this question affirmatively. To explain specific consciousness, many biological theorists hold that each specific type of conscious state corresponds to a specific type of neural state. For instance, Block (2009) hypothesizes that consciousness of motion is simply activation of visual area V5 (as long as this activation is of a certain sort, and is properly embedded in a certain sort of biological system), and similarly Zeki (2007) identifies consciousness of color with activation of V4. This suggests that sensory systems are the neural correlates of specific consciousness. Moreover, Block (2009) speculates that functional connections between these cortical areas and the thalamus are required for consciousness in general, which suggests that sensory and perhaps post-sensory systems (depending on which thalamic regions are involved) are the neural correlates of general consciousness, as well.

Next, consider *higher-order representationalism*, which is a more complex version of first-order representationalism. While first-order representationalists posit an array of first-order perceptual representations directed at the world, higher-order representationalists additionally posit an array of higher-order representations (either thoughts or perceptions) directed at these first-order perceptual representations. To explain general consciousness, higher-order representationalists hold that a state is conscious in virtue of being targeted by (or perhaps being disposed to be targeted by Carruthers, 2000, 2005) higher-order representations. Some higher-order representationalists hold that specific phenomenal character is determined entirely by the content of higher-order representations (Rosenthal, 2005), while others appeal to the content of both first-order and higher-order representations (Lau, 2008, 2010; Kriegel, 2009).

In neural terms, Lau and Rosenthal (2011) hypothesize that higher-order representations are harbored in post-sensory systems, such as posterior parietal regions and especially the dorsolateral prefrontal cortex, while first-order representations are harbored in sensory systems. Although this neural interpretation is not forced on higher-order representationalists, we will henceforth adopt it because (a) several higher-order representationalists have endorsed this interpretation, and (b) higher-order representationalism is very difficult to test scientifically without some neural interpretation. Given this interpretation, higher-order representationalists identify post-sensory areas as the neural correlates of general consciousness; they identify post-sensory and perhaps also sensory areas as the neural correlates of specific consciousness.

Lamme (2006) proposes the *recurrent processing theory*, according to which recurrent processing (as described in §1) is necessary and sufficient for general consciousness. Lamme holds that recurrent processing can occur in both sensory and post-sensory areas. Additionally, while Lamme is not entirely explicit about how to explain specific consciousness, the obvious approach is to say that specific phenomenal character is determined by which areas are engaged in recurrent processing, which again comprises sensory and post-sensory areas.

*Information integration theory* holds that consciousness requires two elements: information, understood as the ability to discriminate among a large number of alternatives, and integration, understood as whether or not a system functions as a unified whole, rather than as separate components (Tononi, 2004, 2008). With respect to general consciousness, information integration theory says that the degree of consciousness that a system possesses at a time is determined by the degree of integrated information it possesses at that time. Since this will, in turn, be determined by corticothalamic networks spanning the brain, the hypothesis takes sensory and post-sensory systems as the neural correlates of general consciousness. Further, with respect to specific consciousness, it claims that the specific character of a conscious state is wholly determined by the set of informational relationships of a system with integrated information (Tononi, 2008). That is, the mechanisms constituting consciousness discriminate one informational state from all other possible states of the system, and this precise discrimination determines the specific phenomenal character of the state. Since all informational relationships are relevant to determining specific phenomenal character, information integration theory also takes sensory and post-sensory systems to constitute specific consciousness.

We now turn to *global workspace theory*, which posits many specialized and localized mental processes. Information from a select few of these processes are collected in a *global workspace* (typically identified with *working memory*), and this information is widely disseminated throughout the brain via long-range neuronal connections. According to global workspace theory, a state is conscious in virtue of its outputs being disseminated via the global workspace, which is neurally realized in cortical and thalamocortical networks distributed throughout the brain but which is especially dense in prefrontal, cingulate, and parietal regions (Baars, 2002, 2005; Baars and Franklin, 2003; Dehaene and Changeux, 2003, 2011; Sergent and Dehaene, 2004; Dehaene et al., 2006, 2011). While these networks consist of post-sensory areas, they relay information from sensory areas. Thus, sensory and post-sensory systems are identified as the neural correlates of general consciousness. Global workspace theory also implicates these same systems as the neural correlates of specific consciousness, since specific phenomenal character is determined by which neurons are active and which are inactive within the global workspace (Dehaene et al., 2011).

Finally, let us return to *first-order representationalism*. Recall that, according to this theory, consciousness is hypothesized to consist in (i) first-order representations directed at the world which (ii) are directly available to the subject for action selection, belief formation, planning, etc. Condition (i) embodies an approach to specific consciousness: the specific phenomenal character of a representation is determined wholly by its content. Condition (ii) complements this with an approach to general consciousness: for a representation to be conscious rather than unconscious is for it to be directly available to the subject for action selection, belief formation, planning, etc.

We will now provide a neural framework for this theory. As mentioned above, we deem arousal or the capacity for arousal (as mediated primarily by subcortical structures in the brainstem, diencephalon and basal forebrain) as a necessary precondition for consciousness, rather than a constitutive factor. Further, with respect to (i), we hypothesize (in agreement with Lau and Rosenthal, 2011) that these first-order representations are generated by sensory systems. In visual processing, for example, we hypothesize (with theorists like Block and Zeki) that V4 contains representations of color and V5 contains representations of motion.

To characterize direct availability neurally, we incorporate elements of global workspace theory. Recall that global workspace theory posits a process whereby information is selected and widely distributed throughout the brain via post-sensory long-range neurons especially dense in prefrontal, cingulate, and parietal regions. (Based on research discussed below, we hypothesize that posterior parietal cortex plays an especially important role.) This process of information selection and distribution integrates such diverse brain functions as attention, long-term memory, motor control, and evaluation (Dehaene et al., 2011). We tentatively hypothesize that the subject is constituted by the integrated activity of such diverse brain functions. Thus, we hold that these post-sensory cortical systems, and the relevant thalamic matrix neurons, underlie direct availability to the subject and are thus the neural correlates of general consciousness. This version of first-order representationalism is a close cousin of global workspace theory with respect to general consciousness, but diverges from global workspace theory with respect to specific consciousness. **Table 1** summarizes this information.

**Table 1:** Theories of consciousness.

Theory	Consciousness is…	Systems constituting general NCC	Systems constituting specific NCC
Biological theory	Identical to neural states	Sensory, possibly post-sensory	Sensory
Higher-order representationalism	Activation of higher-order representations directed at first-order sensory representations	Post-sensory	Post-sensory, possibly sensory
Recurrent processing theory	Recurrent processing	Sensory, post-sensory	Sensory, post-sensory
Information integration theory	Integrated information	Sensory, post-sensory	Sensory, post-sensory
Global workspace theory	Global information availability	Sensory, post-sensory	Sensory, post-sensory
First-order representationalism	Activation of first-order representations (poised to be) distributed via the global workspace	Post-sensory	Sensory

*NCC, neural correlates of consciousness.*

There is one substantial complication, which we mention only to set aside. Some argue that consciousness can overflow access (Block, 1995, 2007, 2011; Lamme, 2003; Tye, 2010), while others disagree (Prinz, 2000, 2005, 2010, 2011; Dehaene et al., 2006; Kouider et al., 2007, 2010; De Brigard and Prinz, 2010; Cohen and Dennett, 2011; Phillips, 2011; Brown, 2012). We remain neutral on this controversial topic, since first-order representationalism is compatible with either view. If consciousness does overflow access, then first-order representationalists can accommodate this by holding that first-order representations can be conscious merely in virtue of being poised for distribution via the global workspace, even if they are not actually distributed. If consciousness does not overflow access, then consciousness can be understood straightforwardly as distribution via the global workspace.

Since only first-order representationalism identifies post-sensory systems as the neural correlates of general consciousness and sensory systems as the neural correlates of specific consciousness, first-order representationalism makes testable predictions distinguishing it from the alternative theories discussed. It predicts that selective impairment of post-sensory systems, such as prefrontal cortex, posterior parietal cortex, and regions of the thalamus, will result in an impaired ability to generate conscious states at all, but that no such impairment will result from impairments of sensory systems alone. These predictions separate first-order representationalism from biological theory, recurrent processing theory, information integration theory, and global workspace theory. Second, it predicts that selectively impairing sensory systems will selectively impair the ability to experience specific types of conscious states without impairing the ability to experience other types of conscious states. Selectively impairing post-sensory systems will not have this effect. These predictions separate first-order representationalism from higher-order representationalism, recurrent processing theory, information integration theory, and global workspace theory.

Next, §3 will argue that the first-order representationalist predictions about general consciousness are consistent with current data, and §4 will argue that the first-order representationalist predictions about specific consciousness are consistent with current data.

## 3. GENERAL CONSCIOUSNESS

The claim that the neural correlates of general consciousness include post-sensory systems in prefrontal cortex, posterior parietal cortex, and non-specific thalamic nuclei is strongly supported by many types of studies. First, consider data suggesting that sensory processing alone appears insufficient for general consciousness. Boveroux et al. (2010) found preservation of functional networks subserving primary visual and auditory processing during deep sedation. Meanwhile, prefrontal cortex (especially dorsolateral prefrontal cortex) appears relevant to general consciousness under some conditions. For example, Lau and Passingham (2006) used a metacontrast masking paradigm to create two conditions with matched performance of discriminating two targets, but where subjects in one condition were much more likely to report seeing the target. fMRI data revealed differences between these conditions only in dorsolateral prefrontal cortex (and not, for example, in sensory processing areas). Additionally, transcranial magnetic stimulation applied to dorsolateral prefrontal cortex alone can decrease the likelihood that subjects report seeing a target (Rounis et al., 2010). Because of the interventional nature of transcranial magnetic stimulation, this suggests that dorsolateral prefrontal cortex is not merely a neural consequence of general consciousness, but is instead a genuine neural correlate. The fact that general consciousness does not wholly disappear under disruption of the dorsolateral prefrontal cortex suggests that this area is not the sole neural correlate of general consciousness.

Långsjö (2012) and Ku et al. (2011) assessed loss and return of consciousness in anesthetized subjects. Loss of consciousness was associated with decreased frontoparietal connectivity (Ku et al., 2011), while return of consciousness was associated with an increase in frontoparietal connectivity (Ku et al., 2011; Långsjö, 2012). Notably, these results also implicate the posterior parietal cortex in general consciousness. However, it is unlikely that prefrontal cortex is the sole neural correlate of general consciousness, given the well-known suppression of frontal activity during rapid-eye-movement (REM) sleep, which is strongly associated with dream consciousness (Maquet et al., 1996; Hobson and Pace-Schott, 2002).

The thalamus also plays a substantial role in supporting general consciousness. Alkire and Miller (2005) report that, across eight different anesthetic agents, depression of the thalamus is more strongly correlated with anesthesia than local effects anywhere else in the brain. Further, recent data suggest that anesthetic effects may preferentially inhibit the non-specific thalamic nuclei and that this inhibition correlates best with cognitive changes during loss of consciousness in humans (Liu et al., 2013). This is consistent with our view that the non-specific thalamic nuclei are part of a post-sensory processing system that mediates general consciousness.

Recall that our neural version of first-order representationalism holds that general consciousness requires availability of sensory content to a set of distributed post-sensory systems (realized in prefrontal cortex, posterior parietal cortex, and thalamus) working in an integrated fashion (as described by global workspace theory). If this is correct, then perhaps no single post-sensory system is required for general consciousness. Rather, integrated activity of a substantial number of post-sensory systems is required for general consciousness. This hypothesis explains the above data concerning deactivation of prefrontal cortex during sleep and is further supported by a number of experiments. For example, Massimini et al. (2010) observed a pattern of widespread cortical activation associated with transcranial magnetic stimulation in the waking state. This pattern persisted, though less robustly, in REM sleep (associated with dreaming consciousness) and was virtually extinguished during non-REM (NREM) sleep (associated with little to no consciousness) and during general anesthesia (Ferrarelli et al., 2010). Similarly, Del Cul et al. (2007) found that consciousness in a masking paradigm was associated with highly distributed fronto-parieto-temporal activation. Such data underscore the significance of widespread cortical activation for general consciousness.

Moreover, not all post-sensory processing is conscious. For example, inhibitory control is mediated largely by the prefrontal cortex, a paradigmatic post-sensory region. Recent studies suggest that inhibitory control can occur unconsciously (Hughes et al., 2009; van Gaal et al., 2008, 2009, 2010, 2011; van Gaal and Lamme, 2011). Similarly, prefrontal cognitive control functions such as task-set preparation may be discharged unconsciously (Lau and Passingham, 2007). As first-order representationalists predict, there was no widespread activation of cortical networks associated with the global workspace in these experiments. Only localized activation of post-sensory regions occurred (Dehaene and Changeux, 2011; van Gaal and Lamme, 2011).

One might argue that, rather than subserving consciousness *per se*, post-sensory processing merely subserves attention or access to sensory systems, which are in fact the neural correlates of general consciousness. On this view, post-sensory processing is a neural consequence of consciousness. One argument for this is neural: Lamme (2003) suggests that the neural basis of attention involves increased activity in frontoparietal regions which is shaped by a combination of sensory processing, short-term memory, and long-term memory. By contrast, he argues that consciousness requires recurrent processing. Backward masking, which renders stimuli unreportable, suppresses recurrent processing while leaving the feedforward sweep largely intact (Lamme and Roelfsema, 2000). Moreover, transcranial magnetic stimulation that disrupts recurrent but not feedforward processing still leaves subjects unable to report seeing anything, and feedforward processing persists without recurrent processing in anesthetized animals (Lamme, 2003). Thus, Lamme concludes that the neural correlates of consciousness include recurrent processing. Although Lamme focuses primarily on recurrent processing within the visual modalities, others theorize that more global recurrent processing from anterior to posterior post-sensory brain regions is critical for general consciousness (Dehaene and Changeux, 2011; Changeux, 2012).

We reply that first-order representationalism is perfectly compatible with these data: as discussed in the next section, the neural correlates of specific consciousness may well include recurrent processing. However, some preliminary evidence argues against the hypothesis that the sole contribution of post-sensory processing is to enable attention or access. For example, Del Cul et al. (2009) studied patients with frontal lesions and found that such patients displayed impaired abilities to report seeing masked stimuli and to select the appropriate response in a forced-choice identification task, compared to control subjects. However, this appears not to be an attentional deficit, for such patients benefited from both temporal and spatial cues, and the threshold for reportable perception was consistently higher for these patients across four different attentional conditions.

More studies are required to rule out the hypothesis that the sole contribution of post-sensory processing is to enable non-attentional access. Nevertheless, the simplest explanation of the current data is that, assuming cortical arousal (as in the waking state and during REM sleep), post-sensory processing regions *alone* are the neural correlates of general consciousness. This result favors first-order representationalism and higher-order representationalism, which both predict this result. It undermines biological theory, which (as it has been specified by its advocates) identifies sensory (and perhaps also post-sensory) processing regions as the neural correlates of general consciousness. It also undermines information integration theory and global workspace theory, which identify both sensory and post-sensory processing regions as the neural correlates of general consciousness. The next section considers specific consciousness.

## 4. SPECIFIC CONSCIOUSNESS

A large body of experimental findings supports the hypothesis that specific phenomenal character is determined solely by sensory systems. The best evidence for this claim is that activation of localized sensory areas is tightly associated with specific conscious states. For example, many studies show that activation of V4 is strongly associated with consciousness of color: fMRI data show selective activation of V4 in the experience of color across a variety of paradigms (Bartels and Zeki, 2000), and damage to V4 leads to the inability to see color (Zeki, 1990). Similarly, recent studies have associated distinct sensory areas with the processing of texture, color, shape/form, and glossiness (Cant et al., 2009; Cavina-Pratesi et al., 2010a, b; Kentridge et al., 2012). Additionally, many studies suggest that activation of V5 is tightly associated with consciousness of motion. Lesions of V5 in humans and monkeys result in severe deficits in motion perception (Schenk and Zihl, 1997). Further, V5 is differentially activated when subjects experience illusory motion (Zeki et al., 1993) and implied motion (e.g., when one sees a still photo of an object obviously in motion; Kourtzi and Kanwisher, 2000).

Surprisingly, such results hold up even across modalities. Saenz et al. (2008) provide fMRI data for early-blind subjects who recovered limited vision in adulthood. In these cases, V5 was differentially activated by moving visual and auditory stimuli. Moreover, Poirier et al. (2006) report similar results based on fMRI data: areas typically associated with visual processing of motion are, in blind subjects, associated with *auditory* processing of motion. Additionally, Ricciardi et al. (2011) found that selectively disabling V5 with transcranial magnetic stimulation significantly impaired the ability of subjects to detect *tactile* motion.

However, some experiments suggest that V5 activation in the absence of V1 activation does not result in consciousness of motion (Cowey and Walsh, 2000; Pascual-Leone and Walsh, 2001). Thus, we leave open the possibility that consciousness of motion requires recurrent processing between V5 and areas such as V1. More broadly, we leave open the possibility that all specific consciousness requires recurrent processing (as Lamme, 2006, 2010 suggest).

Nonetheless, two alternative views are worth considering. First, one may hold that these data concern unconscious or pre-conscious processing, rather than full-fledged conscious processing (Dehaene et al., 2006). On this view, differences in sensory processing are neural prerequisites of consciousness: they cause differences in post-sensory processing, but only these post-sensory states are genuinely conscious. Second, one may argue that some specific phenomenal character is determined by sensory processing, while other specific phenomenal character is determined by post-sensory processing (Lau, 2008, 2010). However, the same problem afflicts both replies. Both of these approaches predict that selectively disabling post-sensory areas will also selectively disable the ability to undergo specific conscious states (just as disabling V5 also selectively disables the ability to be in the specific conscious states associated with motion). However, so far as we know, no effect of this sort has been reported. As discussed in Section “General Consciousness,” disabling post-sensory systems leads to a general deficit in consciousness. However, clear examples of specific deficits in consciousness resulting from local disruption of post-sensory systems have not been found.

In sum, these data strongly suggest that processing in sensory systems alone is the neural correlate of specific consciousness. This result is predicted by first-order representationalism and biological theory but poses problems for higher-order representationalism, information integration theory, and global workspace theory. Since the previous section argued that current forms of the biological theory are unpromising with respect to general consciousness, we conclude that a diverse body of data presently supports first-order representationalism.

## CONCLUSION

We have argued that disentangling general and specific consciousness leads to substantial progress: post-sensory systems are strong candidates for the neural correlates of general consciousness, while sensory systems are strong candidates for the neural correlates of specific consciousness. Of existing theories of consciousness that supply enough neural detail to be directly empirically testable, only first-order representationalism makes these predictions.

However, we freely admit that rival theories of consciousness could be substantially modified to accommodate these data. For example, one could hold a higher-order representationalist view according to which a state is conscious if and only if it is the target of a higher-order representation, but specific phenomenal character is determined solely by the content of the targeted first-order representation. Similarly, a biological theorist could posit that prefrontal cortex, posterior parietal cortex, and non-specific thalamic nuclei constitute general consciousness. It remains to be seen whether such modifications can be made while respecting the initial motivations for these theories. Regardless, we conclude that, of the major theoretical options* as they have been neurally specified by their advocates*, first-order representationalism currently best fits the data.

## Conflict of Interest Statement

The authors declare that the research was conducted in the absence of any commercial or financial relationships that could be construed as a potential conflict of interest.
